# Interaction of the Emerging Mycotoxins Beauvericin, Cyclopiazonic Acid, and Sterigmatocystin with Human Serum Albumin

**DOI:** 10.3390/biom12081106

**Published:** 2022-08-11

**Authors:** Eszter Fliszár-Nyúl, Zelma Faisal, Renáta Skaper, Beáta Lemli, Bayarsaikhan Bayartsetseg, Csaba Hetényi, Patrik Gömbös, András Szabó, Miklós Poór

**Affiliations:** 1 Department of Pharmacology, Faculty of Pharmacy, University of Pécs, Rókus u. 2, H-7624 Pécs, Hungary; 2 Food Biotechnology Research Group, János Szentágothai Research Centre, University of Pécs, Ifjúság útja 20, H-7624 Pécs, Hungary; 3 Department of Organic and Pharmacological Chemistry, Faculty of Pharmacy, University of Pécs, Szigeti út 12, H-7624 Pécs, Hungary; 4 Green Chemistry Research Group, János Szentágothai Research Centre, University of Pécs, Ifjúság útja 20, H-7624 Pécs, Hungary; 5 Pharmacoinformatics Unit, Department of Pharmacology and Pharmacotherapy, Medical School, University of Pécs, Szigeti út 12, H-7624 Pécs, Hungary; 6 Institute of Physiology and Nutrition, Department of Physiology and Animal Health, Agribiotechnology and Precision Breeding for Food Security National Laboratory, Hungarian University of Agriculture and Life Sciences, H-2103 Gödöllő, Hungary

**Keywords:** beauvericin, cyclopiazonic acid, sterigmatocystin, human serum albumin, albumin–ligand interaction

## Abstract

Beauvericin (BEA), cyclopiazonic acid (CPA), and sterigmatocystin (STC) are emerging mycotoxins. They appear as contaminants in food and animal feed, leading to economic losses and health risks. Human serum albumin (HSA) forms stable complexes with certain mycotoxins, including ochratoxins, alternariol, citrinin, and zearalenone. HSA binding can influence the toxicokinetics of xenobiotics, and albumin can also be considered and applied as a relatively cheap affinity protein. Therefore, we examined the potential interactions of BEA, CPA, and STC with HSA employing fluorescence spectroscopy, ultracentrifugation, ultrafiltration, and molecular modeling. Spectroscopic and ultracentrifugation studies demonstrated the formation of low-affinity BEA–HSA (*K_a_* ≈ 10^3^ L/mol) and moderately strong CPA–HSA and STC–HSA complexes (*K_a_* ≈ 10^4^ L/mol). In ultrafiltration experiments, CPA slightly displaced each site marker (warfarin, naproxen, and camptothecin) tested, while BEA and STC did not affect significantly the albumin binding of these drugs. Modeling studies suggest that CPA occupies Sudlow’s site I, while STC binds to the Heme site (FA1) on HSA. Considering the interactions of CPA with the site markers, the CPA–HSA interaction may have toxicological importance.

## 1. Introduction

**Figure 1:**
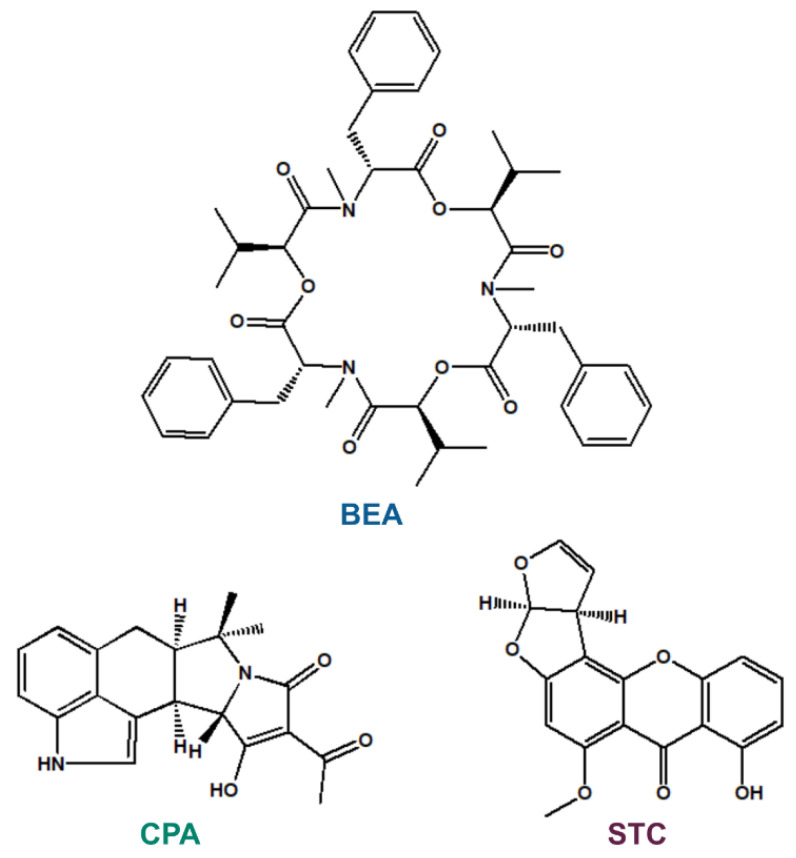
Chemical structures of beauvericin (BEA), cyclopiazonic acid (CPA), and sterigmatocystin (STC).

Mycotoxins appear as contaminants in several food products and animal feed, leading to economic losses and health risks [1]. Beauvericin (BEA; Figure 1) is an emerging mycotoxin with a cyclic hexadepsipeptide structure. It was first isolated from the insect-pathogenic fungus *Beauveria bassiana* [2], but it is also produced by *Fusarium* species [3]. BEA predominantly contaminates grains [4]. It is a potent inhibitor of the cholesterol acyltransferase enzyme; affects the transmembrane transport of mono- and divalent cations (e.g., K^+^ and Ca^2+^); and displays antibacterial, insecticidal, antiviral, and antifungal effects [5,6,7]. BEA has low acute toxicity; however, the lack of data does not allow appropriate risk assessment in regard to its chronic exposure [7,8]. Nowadays, BEA is also widely examined as a potential anticancer agent [6].

Cyclopiazonic acid (CPA; Figure 1) is produced by *Penicillium* and *Aspergillus* species. It typically contaminates peanuts, maize, figs, rice, tomato products, and cheese [9,10]. The acute toxicity of CPA is considered to be low; however, chronic exposure can cause degenerative and other toxic (e.g., nephrotoxic, hepatotoxic, and neurotoxic) effects, showing large variations in different species [9]. The neurotoxic impacts of CPA resemble the toxic side effects of the antipsychotic drugs chlorpromazine and reserpine in mice and rabbits [11].

Sterigmatocystin (STC; Figure 1) is a polyketide secondary metabolite of *Aspergillus* species. It occurs in grains, coffee beans, cheese, spices, and soy beans [12]. STC is a biogenic precursor of aflatoxin B1 synthesis; therefore, the toxic effects of these two mycotoxins are similar [12,13]. The acute toxicity of STC is relatively low; however, even acute exposure can cause hepatocellular necrosis in certain species, and it is nephrotoxic in rats and monkeys [14]. In addition, genotoxic effects of STC are also suggested by in vitro studies [15].

Human serum albumin (HSA) is a plasma protein (66.5 kDa) that is essential for maintaining the oncotic pressure of the blood [16]. Furthermore, HSA forms highly stable complexes with a wide variety of endogenous and exogenous compounds, which can strongly affect their pharmacokinetic or toxicokinetic properties [17]. The major binding sites of drugs and xenobiotics are Sudlow’s site I (subdomain IIA), Sudlow’s site II (subdomain IIIA), and Heme site (or FA1; subdomain IB) on HSA [16,18]. Strong interactions of certain mycotoxins with HSA have been reported, including ochratoxins (*K_a_* ≈ 10^6^ to 10^7^ L/mol) [19,20], alternariol (*K_a_* = 4 × 10^5^ L/mol) [21], citrinin (*K_a_* = 2 × 10^5^ L/mol) [22], and zearalenone (*K_a_* = 10^5^ L/mol) [23]. However, we did not find data in regard to the albumin binding of BEA, CPA, or STC in the scientific literature. Besides the potential toxicokinetic importance of mycotoxin–albumin complexation, serum albumin can also be considered as an affinity protein. Therefore, albumin has been successfully applied for the extraction and purification of the mycotoxins ochratoxin A and alternariol from aqueous solutions, including wine samples [24,25,26].

In this study, we aimed to investigate the possible complex formation of BEA, CPA, and STC with HSA. Toxin–albumin interactions were examined employing fluorescence spectroscopy, ultracentrifugation, ultrafiltration, and molecular modeling. Our results demonstrate that CPA and STC bind to HSA with moderately strong affinity, while BEA forms only poorly stable complexes with the protein.

## 2. Materials and Methods

### 2.1. Reagents

Beauvericin (BEA) was purchased from Cfm Oskar Tropitzsch GmbH (Marktredwitz, Germany). Cyclopiazonic acid (CPA), sterigmatocystin (STC), human serum albumin (HSA), racemic warfarin, racemic naproxen, and S-camptothecin were obtained from Merck (Darmstadt, Germany). Other reagents applied were of analytical or HPLC grade.

### 2.2. Fluorescence Spectroscopic Studies

Fluorescence spectra were recorded using a F-4500 fluorescence spectrophotometer (Hitachi, Tokyo, Japan), while UV–vis spectra were collected employing a V730 UV–vis spectrophotometer (Jasco, Tokyo, Japan). Spectroscopic studies were performed in phosphate-buffered saline (PBS, pH 7.4) at 25 °C. The inner filter effect of mycotoxins was corrected as described previously [21,27]:

(1) $$I_{cor}=I_{obs}\times e^{\left(A_{ex}+A_{em}\right)/2}$$

where *I_cor_* is the corrected fluorescence emission intensity at 340 nm, *I_obs_* is the observed fluorescence emission intensity at 340 nm, *A_ex_* is the absorbance of the mycotoxin at 295 nm, and *A_em_* is the absorbance of the mycotoxin at 340 nm.

In the absence and presence of mycotoxins tested (0–10 μM), the fluorescence emission spectra of HSA (2 μM) were recorded, applying a 295 nm excitation wavelength. The effects of BEA, CPA, and STC on the emission signal of albumin were evaluated at 340 nm employing the Stern–Volmer equation (linear fitting) [23,28]:

(2) $$\frac{I_{0}}{I}=1+K_{SV}\times \left[Q\right]$$

where *I* and *I*_0_ are the emission intensities of HSA with and without the mycotoxins examined, respectively; while *K_SV_* (with the unit of L/mol) is the Stern–Volmer quenching constant, and [*Q*] is the molar concentration (with the unit of L/mol) of the quencher. Thereafter, the association constants (*K_a_*) of mycotoxin–HSA complexes were also determined by employing the Hyperquad2006 program (non-linear fitting) as described earlier [23,28].

### 2.3. Ultrafiltration Studies

Binding sites on HSA as well as the displacing ability of BEA, CPA, and STC vs. site markers were tested with ultrafiltration, as described previously [21,29]. Briefly, before ultrafiltration, samples contained warfarin and HSA (1.0 and 5.0 μM, respectively), naproxen and HSA (1.0 and 1.5 μM, respectively), or camptothecin and HSA (1.0 and 1.5 μM, respectively) without and with mycotoxins (20 μM) in PBS (pH 7.4). Samples were centrifuged (10 min, 7500× *g*, 25 °C; fixed-angle rotor) in Pall Microsep Advance centrifugal devices with 30 kDa molecular weight cutoff (MWCO) value (VWR, Budapest, Hungary). After centrifugation, the concentrations of the site markers in the filtrates were determined with HPLC (see Section 2.5).

### 2.4. Ultracentrifugation Experiments

To confirm the results of quenching studies, the free (not albumin-bound) fraction of mycotoxins was determined in the presence of albumin. Ultracentrifugation under the proper conditions makes possible the sedimentation of the protein without the disruption of albumin–ligand interactions [29,30]. Thus, the free fraction of the ligand molecule can be determined in the supernatant. Since the typical plasma level of HSA in the circulation is 35–50 g/L, samples contained 40 g/L (≈600 μM) of HSA and 10 μM concentration of the mycotoxins tested (BEA, CPA, or STC) in PBS (pH 7.4). A 900 μL aliquot of samples was transferred into 11 × 34 mm PC tubes (Beckman Coulter, Brea, CA, USA) and centrifuged for 16 h at 170,000× *g* and 20 °C, employing a Beckman Coulter Optima MAX-XP tabletop ultracentrifuge (with an MLA-130 fixed-angle rotor). Thereafter, a 200 μL fraction of the upper part of the protein-free supernatant was carefully removed. BEA and CPA were directly analyzed, while STC solutions were acidified to pH 5 with 1 M HCl before HPLC analysis (see details in Section 2.5).

To validate the ultracentrifugation method, the same experiments were also performed with warfarin and paracetamol. Warfarin forms highly stable complexes with HSA; therefore, approximately 99% of it is albumin-bound in the human circulation [31]. On the other hand, paracetamol has only a weak interaction with the protein, and its albumin-bound fraction is less than 20% in the blood [32]. After ultracentrifugation, warfarin and paracetamol were directly analyzed from the supernatants with HPLC (see details in Section 2.5).

Assuming the 1:1 stoichiometry of complex formation, we determined association constants (*K_a_*) using the following equation [33]:

(3) $$K_{a}=\frac{\left[LP\right]}{\left[L\right]\times \left[P\right]}$$

where [*L*], [*P*], and [*LP*] are the molar concentrations of the unbound free ligand, the unbound free protein, and the ligand–protein complex, respectively.

### 2.5. HPLC and LC-MS Analyses

For HPLC-UV and HPLC-FLD analyses, an integrated HPLC system (Jasco, Tokyo, Japan) was used, it was built up from an autosampler (AS-4050), a binary pump (PU-4180), a fluorescence detector (FP-920), and an UV detector (UV-975). Chromatographic data were evaluated employing ChromNAV2 software (Jasco). The concentrations of warfarin and camptothecin were determined by HPLC-FLD, while naproxen was quantified by HPLC-UV using the previously described methods without modification [21,29]. CPA and STC were quantified employing the recently reported HPLC-UV methods [34].

Concentrations of paracetamol were determined by HPLC-UV. A 20 μL volume of samples was driven through a guard column (SecurityGuard Cartridge, Phenyl 4.0 × 3.0 mm; Phenomenex, Torrance, CA, USA) linked to a Kinetex Phenyl–Hexyl 100A analytical column (150 × 4.6 mm, 5 μm; Phenomenex). The isocratic elution was carried out with a 1.0 mL/min flow rate at room temperature, employing a potassium phosphate buffer (10 mM, pH 4.5) and acetonitrile (92:8 *v*/*v*%) as the mobile phase. Paracetamol was detected at 245 nm.

Concentrations of BEA in the supernatants were determined with a Shimadzu 2020 LC-MS system (Shimadzu, Kyoto, Japan). To obtain high-resolution chromatographic separation, a XB-C18 Kinetex analytical column (100 × 2.1 mm, 2.6 µm; Phenomenex) was used with a 0.3 mL/min flow rate (injected sample volume: 10 µL). The gradient elution was performed employing eluents A (0.1% formic acid + 0.005 M ammonium formate) and B (acetonitrile + 0.1% formic acid), using the following gradient program: 0.0–1.0 min 5% eluent B, 1.0–3.0 min linear increase of eluent B to 60%, 3.0–4.0 min 60% eluent B, 4.0–8.0 min linear increase of eluent B to 95%, 8.0–10.9 min 95% eluent B, 10.9–12.5 linear decrease of eluent B to 5%, and 12.5–15 min 5% eluent B. The used *m*/*z* values were 784(+) and 801(+).

### 2.6. Modeling Studies

The 3D structures of the ligand molecules (CPA and STC) were built by CORINA classic [35,36] from their respective SMILES strings, and the resulting structures were downloaded in pdb format. Prior to the structural optimization, OpenBabel [37], a chemical tool box, was employed to add Gasteiger–Marsili partial charges and convert the ligand structures from PDB to MOL2 format. Further, two-step minimization was performed with OpenBabel, including a steepest-descent followed by a conjugate-gradient step. The maximum number of steps was set to 100,000, and the MMFF94 force field was used for all calculations. The pre-optimized structures then underwent semi-empirical quantum mechanics optimization with PM7 parameterization and a gradient norm of 0.001 using MOPAC2016 [38]. Finally, the minimized structures were converted back to PDB files using OpenBabel.

Blind docking was performed with AutoDock4 [39] using the optimized ligand and target structures. The Gasteiger partial charges were assigned to the HSA and ligand structures, and then, the non-polar hydrogens were merged using AutoDock tools. During docking calculations, HSA was considered a rigid body while all the torsional bonds of two ligand structures were taken as flexible. In grid calculations, the grid box size was set to 126 × 100 × 126 with a 0.375 Å grid spacing center, and the grid box was centered on the mass center of HSA. Docking was carried out using the Lamarckian genetic algorithm and the number of docking runs was set to 20 with a maximum number of evaluations of 25 million. During docking, the root-mean-square (RMS) cluster tolerance was set to 2 Å.

### 2.7. Statistics

Data (mean ± SEM) were derived at least from three independent experiments. Statistical evaluation (*p* < 0.05 and *p* < 0.01) was performed by one-way ANOVA (and Tukey post hoc) test using SPSS Statistics software (IBM, Armonk, NY, USA).

## 3. Results and Discussion

### 3.1. Fluorescence Quenching Studies

**Figure 2:**
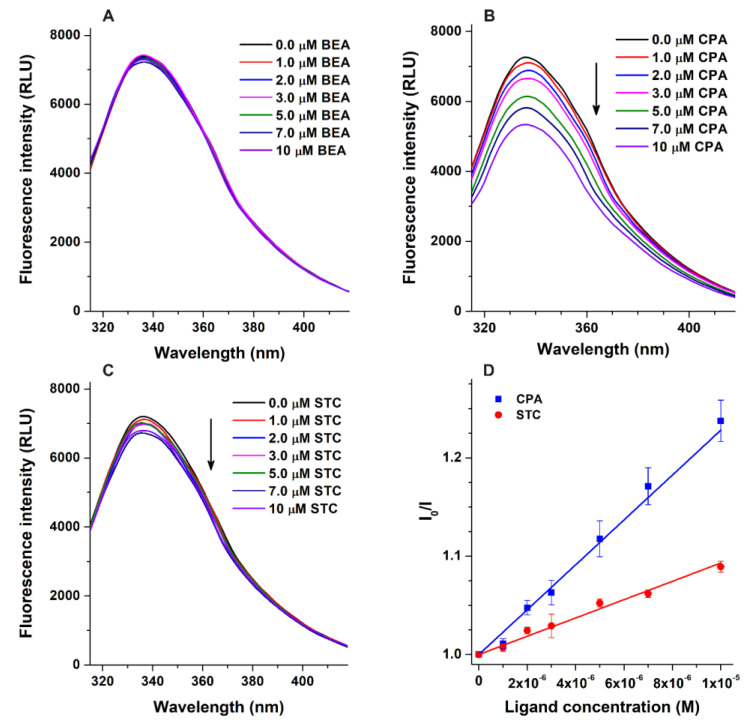
Fluorescence emission spectra of HSA (2.0 μM) in the presence of the increasing concentrations of BEA (**A**), CPA (**B**), and STC (**C**) in PBS (pH 7.4; λ_ex_ = 295 nm). Stern–Volmer plots of CPA–HSA and STC–HSA complexes (**D**; λ_ex_ = 295 nm, λ_em_ = 340 nm).

The fluorescence quenching effects of BEA, CPA, and STC on HSA (2 μM) were tested in the presence of increasing mycotoxin concentrations (0–10 μM). Trp-214 (located in site I, subdomain IIA) is mainly responsible for the fluorescence of HSA, while tyrosine and phenylalanine amino acids have much lower importance from this point of view [16]. The size of HSA is not very large (66.5 kDa); therefore, the interaction of ligands with this macromolecule typically modifies the emission signal of the protein, even if the binding site is not located in Sudlow’s site I. Under the applied conditions, the emission signal of HSA at 340 nm was barely affected by BEA (Figure 2A), suggesting that this mycotoxin does not form or forms poorly stable complexes with the protein. Furthermore, after the correction of the inner-filter effect, CPA and STC caused large and slight quenching effects on HSA, respectively (Figure 2B,C). If the binding site of a ligand molecule is relatively close to the Trp-214 amino acid (e.g., in site I), then the quenching effect of the ligand is large, while a farther binding position typically results in lower changes in the fluorescence of HSA.

Stern–Volmer plots (Figure 2D) showed good linearity (R^2^ = 0.995 and 0.984 for CPA and STC, respectively). The decimal logarithmic values of Stern–Volmer quenching constants (log*K_SV_*) and association constants (log*K_a_*) are demonstrated in Table 1. The log*K_SV_* values of the CPA–HSA and STC–HSA complexes were similar; however, the log*K_a_* values suggest that CPA binds with slightly (2.5-fold) higher affinity to HSA than STC.

### 3.2. Ultracentrifugation Studies

The quantitative data determined for warfarin (bound fraction: 99.05 ± 0.02%; log*K_a_* = 5.25 ± 0.01) and paracetamol (bound fraction: 16.26 ± 0.15%; log*K_a_* = 2.51 ± 0.01) as reference ligands showed excellent correlation with the previously reported results [31,32], confirming the suitability of the ultracentrifugation method applied. These studies demonstrated that BEA could interact with the protein; however, in agreement with the quenching studies (Figure 2), only poorly stable BEA–HSA complexes were formed (Table 1). On the other hand, in the presence of 40 g/L (≈600 μM) HSA, we measured approximately 3% and 8% as the free (not albumin-bound) fractions of CPA and STC, respectively. Based on Equation (3), the log*K_a_* values were also calculated, showing similar data to that determined in quenching studies and also suggesting that the stability of the CPA–HSA complex is approximately 2.5-fold higher compared to that of STC–HSA (Table 1). Thus, both spectroscopic and ultracentrifugation experiments demonstrated the relevant, moderately strong interactions of CPA and STC with the protein, similar to that of aflatoxins (log*K_a_ =* 4.3 to 4.6) [40], patulin (log*K_a_* = 4.1) [20], or phenytoin (log*K_a_* = 4.0) [41].

### 3.3. Ultrafiltration Studies

**Figure 3:**
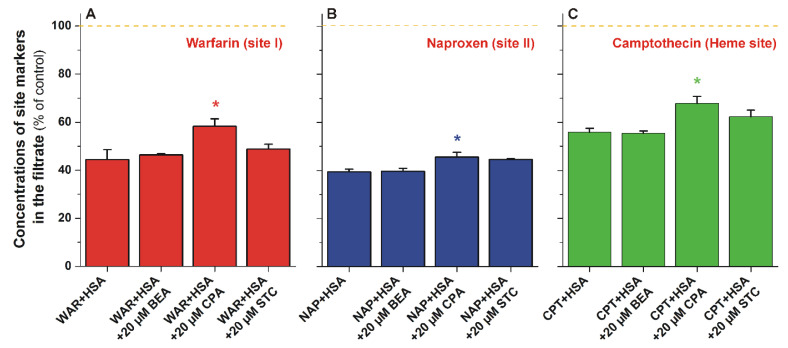
Concentrations of warfarin (**A**), naproxen (**B**), and camptothecin (**C**) in the filtrate after ultrafiltration (MWCO: 30 kDa). Samples contained warfarin and HSA (1.0 and 5.0 μM, respectively) or naproxen and HSA (1.0 and 1.5 μM, respectively), or camptothecin and HSA (1.0 and 1.5 μM, respectively) in the absence and presence of mycotoxins (20 μM) in PBS (pH 7.4; * *p* < 0.05; BEA, beauvericin; CPA, cyclopiazonic acid; STC, sterigmatocystin; WAR, warfarin; NAP, naproxen; CPT, camptothecin; HSA, human serum albumin).

To test the binding sites of mycotoxins on HSA and their potential displacing effects vs. other ligands, ultrafiltration studies were performed employing site I (warfarin), site II (naproxen), and Heme site (camptothecin) markers [21]. HSA and albumin-bound molecules cannot pass through the filter with a 30 kDa MWCO value; therefore, the increased concentration of the site marker in the filtrate indicates its displacement from the protein [21,22]. BEA did not cause any changes (compared to the control) in the filtered fraction of the site markers used (Figure 3), supporting again that BEA does not form stable complexes with HSA. STC induced only minor (not statistically significant) changes vs. the control; while CPA significantly increased the filtered concentrations of each site marker tested, causing a slight elevation for naproxen (6%) and a larger increase for camptothecin (12%) and warfarin (14%) (Figure 3). Site I and the Heme site are allosterically coupled [16], which may explain the similar effects of CPA on site I and the Heme site. The slight displacement of naproxen by CPA may be resulted from its allosteric interaction [41] with the site II marker or may be caused by a potential secondary binding site of the mycotoxin and/or the site marker on HSA [18,42].

### 3.4. Modeling Studies

**Figure 4:**
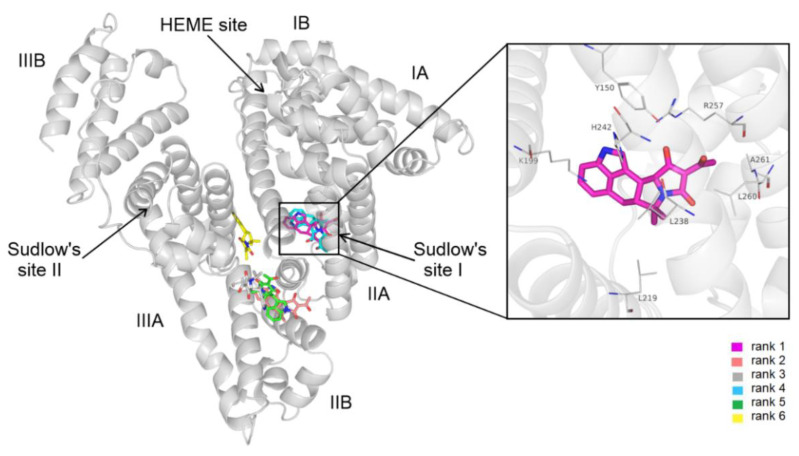
Blind docking results of CPA (sticks colored by docking ranks) on HSA (gray cartoon) with the representative binding positions of different ranks (left panel). In the right panel, a close-up of the binding mode in regard to rank 1 is demonstrated, representing the best energy score in the inset with interacting HSA residues (sticks colored by atom types).

A total of 20 different conformations of CPA were generated through blind docking, and these binding modes were sorted into six clusters based on RMS cluster tolerance between cluster members. After visual inspection, we observed three main binding sites where cluster representatives were located (Figure 4). The first-ranked binding mode was within the hydrophobic binding pocket of Sudlow’s site I. The interaction between HSA and our lowest energy conformation was shown to be equally contributed by hydrogen bonds as well as hydrophobic and cation–π interactions. Hydrophobic interactions are formed with Leu219, Leu238, Arg257, Leu152, and Ala158, while the complex was further stabilized by hydrogen bonds with the main polar residues of the binding site, including Tyr150, Lys199, His242, and Arg257 (Figure 4). The polar residue Lys199 is also shown to be in close proximity with cyclic groups of the CPA, forming cation–π interactions.

Thus, blind docking suggests site I as the high-affinity binding site of CPA on HSA (Figure 4). These data are in agreement with our previous observations: CPA can considerably quench the fluorescence signal of the protein (Figure 2B); furthermore, it can displace the site I ligand warfarin (Figure 3). The displacement of Heme site (camptothecin) and site II (naproxen) markers by CPA may be resulted from allosteric interactions.

**Figure 5:**
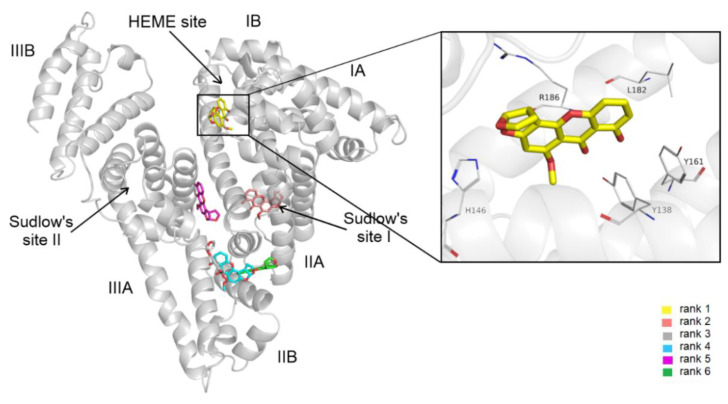
Blind docking results of STC (sticks colored by docking ranks) on HSA (gray cartoon) with the representative binding positions of different ranks (left panel). In the right panel, a close-up of the binding mode in regard to rank 1 is demonstrated, representing the best energy score in the inset with interacting HSA residues (sticks colored by atom types).

The blind docking of STC to HSA resulted in a total 20 of different conformations. Based on cluster tolerance, these binding modes formed six clusters occupying four distinct binding sites on HSA (Figure 5). The lowest energy conformation was found in subdomain IB, also known as FA1 or the Heme site. Subdomain IB has been identified as the primary binding site of bilirubin and hemin on HSA [18]. STC–HSA complexation is shown to be dominated by hydrophobic interactions, the cyclohexyl groups of STC interact with Tyr138, Tyr161, Leu182, and Arg186. Furthermore, cation–π interaction with Arg186 and salt bridge formation with His146 were also noticed by the heterocyclic groups of the ligand (Figure 5). In addition, there is a hydrogen bond between the carbonyl group of STC and the hydroxyl group of Tyr138.

Modeling studies identified the Heme site (rank 1) as the most likely high-affinity binding site of STC on HSA. These data are in agreement with our observation that STC caused only a minor decrease in the fluorescence emission signal of the protein (Figure 2C).

## 4. Conclusions

In this study, the interactions of BEA, CPA, and STC with HSA were examined. Fluorescence quenching, ultracentrifugation, and ultrafiltration studies showed that BEA forms only poorly stable complexes with the protein. However, we noticed moderately strong interactions of CPA and STC with HSA. Importantly, CPA displaced each site marker tested (site I, site II, and Heme site) from albumin, suggesting that the CPA–HSA interaction may have toxicological importance, while STC–HSA complex formation likely has lower biological relevance. Experimental and modeling approaches suggest that the binding site of CPA is located in Sudlow’s site I, while STC likely occupies the Heme site on albumin. Our results provide novel information for the deeper understanding of albumin binding and toxicokinetics of these emerging mycotoxins.

## Figures and Tables

**Table 1:** Decimal logarithmic values of Stern–Volmer quenching constants (*K_SV_*; unit: L/mol) and association constants (*K_a_*; unit: L/mol) of CPA–HSA and STC–HSA complexes based on spectroscopic (see details in Section 2.2) and ultracentrifugation (see details in Section 2.4) experiments; and the bound fraction (%) of mycotoxins in the presence of 40 g/L (≈600 μM) HSA in PBS (pH 7.4; see experimental details in Section 2.4).

Complex	log*K_SV_* ± SEM *FL Quenching* (2 μM HSA)	log*K_a_* ± SEM *FL Quenching* (2 μM HSA)	log*K_a_* ± SEM *Ultracentrifugation* (40 g/L HSA)	Bound Fraction (%) *Ultracentrifugation* (40 g/L HSA)
**BEA–HSA**	–	–	3.19 ± 0.02	47.9 ± 0.9
**CPA–HSA**	4.37 ± 0.05	4.38 ± 0.05	4.70 ± 0.02	96.7 ± 0.2
**STC–HSA**	4.32 ± 0.10	3.98 ± 0.06	4.28 ± 0.04	91.8 ± 0.7

## Data Availability

Not applicable.
